# Some Aspects of the Thermochemical Route for the Valorization of Plastic Wastes, Part I: Reduction of Iron Oxides by Polyvinyl Chloride (PVC)

**DOI:** 10.3390/ma14154129

**Published:** 2021-07-24

**Authors:** Ndue Kanari, Nour-Eddine Menad, Lev O. Filippov, Seit Shallari, Eric Allain, Fabrice Patisson, Jacques Yvon

**Affiliations:** 1 Université de Lorraine, CNRS, GeoRessources, F-54000 Nancy, France; lev.filippov@univ-lorraine.fr (L.O.F.); ericgallain@gmail.com (E.A.); jyvon6355@gmail.com (J.Y.); 2 Waste and Raw Materials and Recycling Unit, Water, Environment Process and Analysis Department, BRGM, 3 Avenue Claude Guillemin, BP 36009, CEDEX, F-45060 Orléans, France; n.menad@brgm.fr; 3 Faculty of Agriculture and Environment, Agricultural University of Tirana, 1029 Tirana, Albania; seitshallari@gmail.com; 4 Université de Lorraine, CNRS, Labex DAMAS, IJL, F-54000 Nancy, France; fabrice.patisson@univ-lorraine.fr

**Keywords:** waste plastic, halogen content, polyvinyl chloride, isothermal treatment, de-chlorination, direct reduction of iron oxides, circular economy

## Abstract

The mass production of synthetic plastics began in the last century and today they have become one of the most abundant man-made materials. The disposal or the beneficiation of end-of-life plastics represent a great challenge for society especially in the case of polyvinyl chloride (PVC). This study is focused on the use of PVC waste as a useful agent for the direct reduction of hematite (Fe_2_O_3_) after a thermal treatment at 300 °C for removing the chlorine contained in PVC. Thermal reduction tests were conducted from 600 °C to 1100 °C with (Fe_2_O_3_ + PVC + clay) pellet mixtures in which clay was used as plasticizing and binder agent of the pellets. The starting samples and treatment residues were analyzed by scanning electron microscopy through energy dispersive spectroscopy (SEM-EDS) and X-ray diffraction (XRD) to monitor the chemical behavior and reactivity of the pellet constituents during their thermal treatment. The stepwise reduction of hematite up to metallic iron was achieved at temperatures approaching 1000 °C, confirming the capability of using PVC waste for the direct reduction of iron oxides.

## 1. Introduction

Although the history of natural plastics is quite old, their chemical synthesis and mass industrial production has widely developed over the past century. With a tonnage of 1.5 million in 1950, global production of plastics has continued to increase reaching 359 million tons in 2018, thus one of the most abundant man-made materials. Europe contributed for 17% while China produced 30% of the total amount [1]. Nowadays, plastics have invaded our contemporary life and are an integral part of our daily life. There are many industries and economic sectors using plastics from packaging, followed by building and construction, production of consumer goods, automobiles, electrical and electronic equipment, and so on. However, at the end of their life, plastics generate a large quantity (especially a large volume) of residual materials that are unfortunately difficult to degrade naturally.

Recycling, energy recovery, and landfill are the three most common routes for managing plastic waste. According to the OECD data [2], 14–18% of waste plastics are recycled, 24% are incinerated, and 58–62% of them are put into landfills. Note that around 19% of plastic waste [3] is mismanaged (uncontrolled landfills) and often dumped in seas and oceans. As noted by Dąbrowska et al. [4], there may be more plastic waste than fish in the oceans after 2050 if no decisive steps are taken to limit the amount of this waste.

The main commercial resins (PET-polyethylene terephthalate; HDPE-high density polyethylene; PVC-polyvinyl chloride; LDPE-low density polyethylene; PP-polypropylene; and PS-polystyrene, among others) of the industrial plastics and their properties were summarized earlier [5,6]. The resin diversity and the integration of plastics in composite materials such as metal-plastic, glass-plastic, and fiber-plastic that are difficult to be disassemble complicate the recycling of end-of-life plastics. However, given strict environmental concerns and increasing public pressure, the landfill rate of plastic waste is expected to be significantly reduced, while the incineration and recycling rates will increase over the coming decades [7].

One of the most problematic cases of plastic waste is polyvinyl chloride (PVC) with a raw chemical formula (C_2_H_3_Cl)_n_ containing a high amount of chlorine (ca. 57 wt% Cl in pure PVC). As PVC is mostly used for non-packaging materials, it is not collected separately with lightweight packaging waste but rather is collected with residual waste that decreases its recyclability. When incinerated with municipal waste at high temperatures, special measures must be applied for the purification and detoxification of the incinerator outlet gases. As reported by Ma et al. [8], the chlorine causes high temperature corrosion and low efficiency in waste-to-energy processing plants. Note that other potentially polluting substances contained in plastic are the bromine-containing flame retardants still used widely to meet the flammability standards of products [9]. Several recent scientific studies addressing the diverse aspects of plastics containing PVC and halogenated flame retardants were reported in the scientific journal *Materials* [10,11,12,13,14,15].

Several selected studies [16,17,18,19,20,21,22] demonstrated the use of plastic wastes and other hydrocarbon-bearing materials as a substitution for current reducing agents in the iron and steel-making industry. It is shown that using individual plastic resins or municipal plastics wastes in the coke oven and blast furnace can lead to a potential reduction in CO_2_ emissions [16]. Similarly, Trinkel et al. [17] and Bürgler and Kieberger [18] stated that plastic wastes can be utilized in blast furnaces as an alternative reducing agent. In a recent review [19], it was noted that partial or complete substitution of pulverized coal by plastic wastes or renewable carbon-bearing materials similar to plastic waste or biomass contribute in mitigating the CO_2_ emission due to its high H_2_ content compared to fossil carbon. Dankwah et al. [21] reported that blends of plastic wastes (HDPE) with metallurgical coke could be used in electric arc furnace (EAF)-steelmaking as a reductant. They reported a better reduction and a lower CO_2_ emission with increasing plastic wastes to coke ratio. Fick et al. [22] stated that a 20% substitution of biomass for coke, around 300 kg of CO_2_-equivalent per ton of pig iron produced, could be applied, representing a reduction of 15% in the total greenhouse gas emissions. Details about carbon-neutral biomass-based and circular rubber or plastics-based carbon sources and their potential to substitute fossil charge or the injection of carbon in the EAF process are summarized by Echterhof [23]. As noted by Patisson and Mirgaux [24], the reduction of iron oxides has been thoroughly studied as evidenced by thousands of papers published over the last centuries.

As mentioned above, the chlorine content of PVC plastics is a drawback and such plastics cannot be directly used for Fe-oxides reduction because a part of the iron will be transformed into chlorides at a high temperature. Recent research reports suggested the de-chlorination of PVC can be performed by hydrothermal methods in an alkaline system [25,26,27] as well as by the thermal route [28,29,30,31]. Their results agreed that HCl is the initial-bearing chlorine product during PVC treatment by dry methods.

It would be interesting to study the low temperature removal of chlorine (de-chlorination) and its recovery from PVC before further material and energetic beneficiation occurs. In this context, this article addresses the use of PVC as an alternative to classical reducing agents for the direct reduction of iron oxides. The proposed original research design is composed of two steps: (i) The first step involves the study of the temperature effect on the de-chlorination degree of the two kinds of PVC (waste and pure PVC) under air atmosphere, (ii) while the second step involves the investigation on the reducing capability of the de-chlorinated PVC for the reduction of hematite (Fe_2_O_3_) at diverse temperatures and reaction times. Temperatures equal to or less than 1100 °C were preferred which is much lower than those observed during the manufacturing of cast iron and steel.

One may note that both steps can be performed successively in the same reactor; thus, the proposed route is more attractive for a larger scale application.

## 2. Materials and Methods

A PVC waste sample issued from electrical cable coatings with a mean particle size of about 80 μm provided by a French manufacturer specialized in the cryogenic grinding of plastics was used for this investigation. It is designated as W-PVC. A sample of pure PVC [9] with an average mean particle size of 100 μm was also used for the comparison purpose of the thermal behavior; it is noted as P-PVC. The hematite sample, having micrometric particles and with a chemical purity higher than 99.5% Fe_2_O_3_, was procured from Merck Chemicals (Eurolab, Fontenay-sous-Bois, France). A kaolin clay sample serving as the appropriate bonding and plasticizing agent for prepared pellets mixtures (PVC + Fe_2_O_3_ + clay) completed the initial sample set.

**Figure 1:**
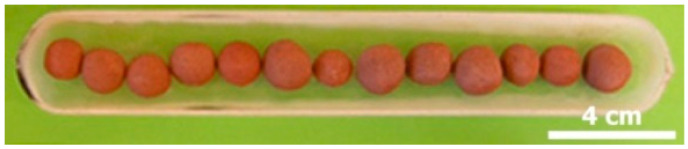
Optical image of the (hematite + W-PVC + clay) pellets mixtures.

For the individual thermal treatment of the three reagents (W-PVC, P-PVC, and clay) at different temperatures, about 5 g (except otherwise indicated) of powder sample was spread out uniformly in a silica boat and used directly. For the thermal process of the PVC-hematite samples, the preparation consisted first in mixing W-PVC, hematite, and clay with water to get a pasty material that was then hand-conditioned as pellets of centimetric size. Hence, the obtained pellets were dried in an oven at 70 °C for 48 h prior to their thermal treatment. A photograph of several pellets composed (35% W-PVC + 35% hematite + 30% clay) inside the experimental quartz boat used for this investigation is displayed in Figure 1.

**Figure 2:**
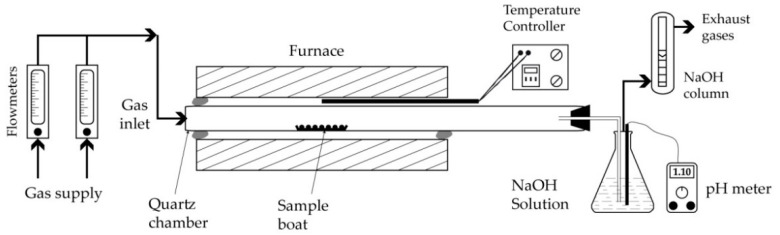
Schematic presentation of the experimental setup.

Experiments to study the thermal behavior of the various samples and their mixtures were performed in the simple experimental setup sketched in Figure 2. The main equipment is a tubular resistance furnace with the ability to reach 1600 °C. The working tube is made of quartz and can withstand temperatures as high as 1300 °C in the absence of corrosive atmospheres for SiO_2_ such as gaseous alkali oxy-hydroxides and fluorides, among others.

In some experiments conducted with W-PVC alone, the outlet gases were scrubbed in a NaOH solution with a defined pH, while the acidity of the solution was recorded during the experiment progress. Other features of the experimental setup included a temperature controller, flowmeters, and a system for the purification of the outlet gases.

Details on the chemical and mineralogical characteristics of the various materials will be provided in the next section. The description of the equipment and analytical protocol used for the physicochemical characterizations was provided elsewhere [32]. Two preparation procedures were applied for the scanning electron microscopy through energy dispersive spectroscopy (SEM-EDS) analysis of the treatment products: (i) The specimen was firstly epoxy-impregnated and after hardening the specimen underwent a polishing step, leading to the smooth surface required for SEM-EDS examination; (ii) the sample was ground while the powdered specimen was glued on a graphite support. In both cases, the samples were coated by a thin layer of carbon to ensure a good electrical conductivity during SEM-EDS examination as this is a handicap for its quantification in the plastic materials.

## 3. Results

### 3.1. Physicochemical Characterization of the Experiment Specimens

**Figure 3:**
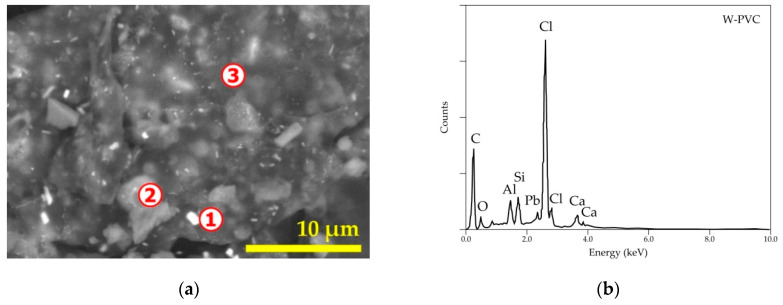
SEM-EDS results of initial PVC waste: (**a**) general view (backscattered electron micrograph “BSE”) of the sample and numbers 1–3 indicate the locations for microanalysis; (**b**) overall EDS spectrum of the sample.

As revealed previously [9] by SEM-EDS analysis, the pure PVC (P-PVC) sample is composed of chlorine and carbon (hydrogen not analyzed by this technique). As aforementioned, the PVC resin has the raw chemical formula (C_2_H_3_Cl)_n_ but other chemicals are added to enhance the functional properties of the bearing PVC materials. A general SEM image and overall EDS spectrum of the PVC waste (W-PVC) are provided in Figure 3. The W-PVC sample (Figure 3b) has a rather multi-elemental composition. Detailed microanalysis as summarized in Table 1 showed the presence of O, Al, Si, Ca, Sb, and Pb as the main PVC constituents apart from chlorine. Lead (spot n° 1 and n° 3) belongs to the lead-based stabilizers for plastic resins, while calcium-bearing compounds distributed throughout the sample are used as the main filler for plastics. Aluminum and silicon oxides are regular constituents of glass fiber-reinforced plastics, while antimony (spot n° 1 and n° 3) is introduced in the plastic for its synergic effect in conjugation with halogens to obtain the fire inhibition property.

**Figure 4:**
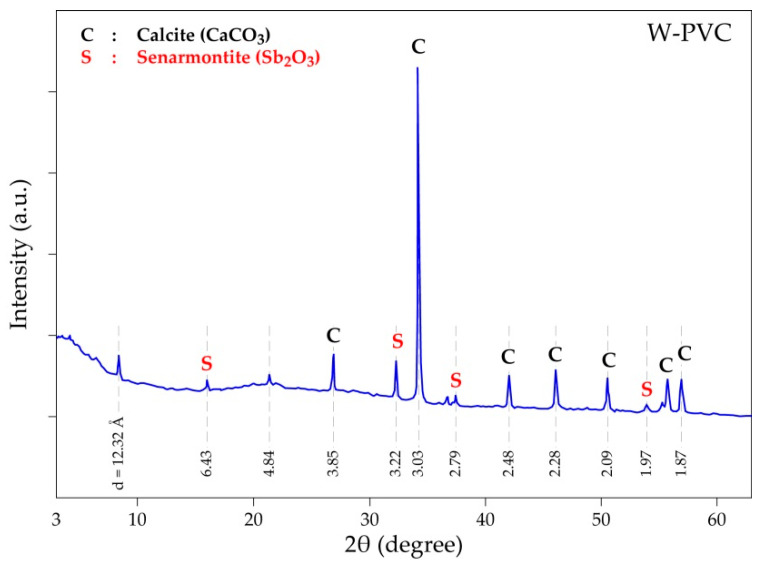
XRD diffractogram of the W-PVC sample.

X-ray diffraction (XRD) patterns of the W-PVC sample (Figure 4) exhibited mostly the characteristic peaks of the crystallized mineral phases such as calcite (CaCO_3_) and senarmontite (Sb_2_O_3_), agreeing fairly with the results of SEM-EDS analyses, while the organic matter seems to have an amorphous nature resulting in several small and broad halos in the X-ray diffractogram of the W-PVC sample.

**Figure 5:**
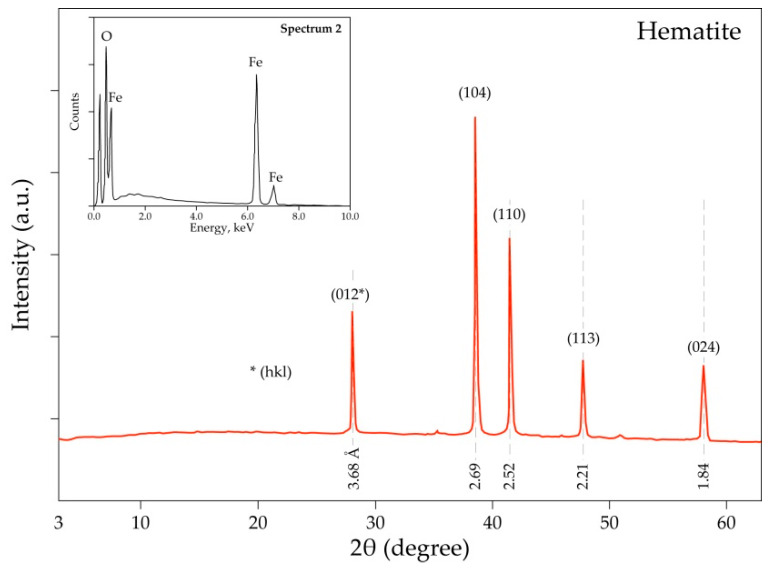
XRD diffractogram and embedded SEM-EDS spectrum (top-left corner) of the hematite sample.

The crystallinity of the hematite sample was determined using XRD and the diffractogram is drawn in Figure 5. It detects the presence of only α-Fe_2_O_3_ as a main phase, confirming the microanalysis performed by SEM-EDS showed in the miniaturized spectrum in Figure 5, as well as the chemical characteristics of the hematite sample provided by the supplier.

**Figure 6:**
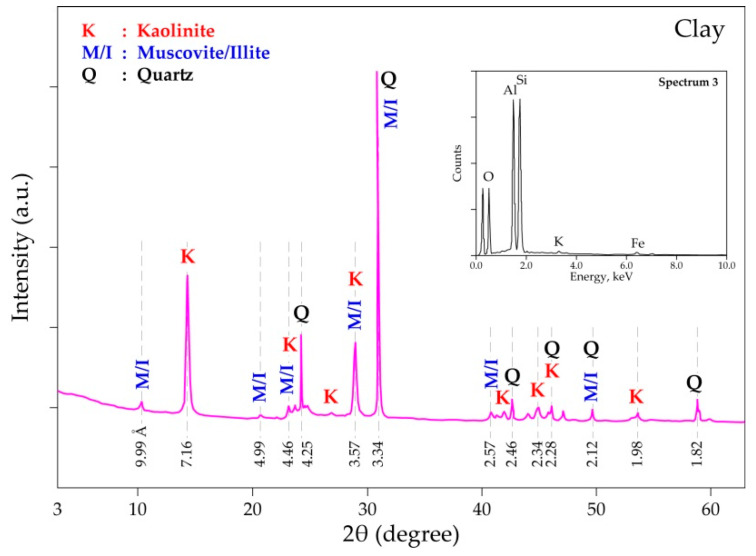
XRD diffractogram and embedded SEM-EDS spectrum (top right corner) of the clay sample.

The qualitative elemental and mineral compositions of the clay sample revealed by SEM-EDS and XRD measurements, respectively, are summarized in Figure 6. The main constituents of the studied clay specimen were kaolinite [Al_2_Si_2_O_5_(OH)_4_], micas {Al_2_[AlSi_3_O_10_(OH,F)_2_K]-muscovite; Al_(2__−x)_(Mg,Fe)_x_Si_3_Al)(K,H_3_O)_x_O_10_[(OH)_2_,(H_2_O)]-illite}, and quartz (α-SiO_2_), while the main elements revealed by SEM-EDS (miniaturized spectrum in Figure 6) consist of Si, Al, O, K, and Fe.

### 3.2. Treatment of the PVC and Clay Samples at Different Temperatures

**Figure 7:**
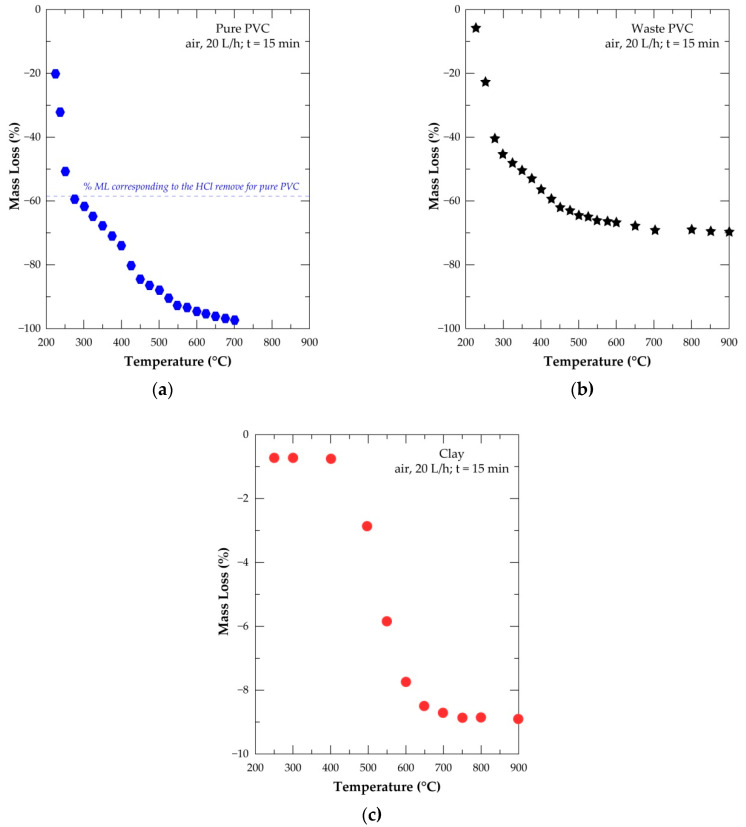
The mass change of the samples versus the temperature for the treatment of samples in air from 200 °C to 900 °C at 15 min: (**a**) pure PVC; (**b**) PVC waste; and (**c**) clay.

The thermal treatment of the two plastics (P-PVC and W-PVC) and clay sample was performed from 225 °C to 900 °C under air with a flow rate of 20 L/h. A short dwelling time (15 min) was chosen in order to better highlight the inherent effect of temperature on the occurring reactions. Results expressed as evolution of % mass loss (%ML) versus temperature for the three samples are grouped in Figure 7. Hematite does not undergo any thermal transformation in this temperature range.

Figure 7a shows that 20% ML was recorded for the P-PVC treated at 225 °C and about 61.5% of the sample was lost at 300 °C. The SEM-EDS analysis of the treatment residue at 300 °C indicated that it is composed only of carbon (hydrogen is not detectable by the SEM-EDS tool). The theoretical mass loss (ML = −56.8%) attributed to the removal of all the chlorine as HCl (dashed line in Figure 7a) from the pure PVC matches well with the experimental percentage of ML of the P-PVC obtained around 275 °C. These evidences in combination with several literature data [28,30] on the de-chlorination of PVC suggested that almost full removal of chlorine from the pure PVC can be achieved around this temperature. The sample mass loss for P-PVC at higher temperatures is due to the subsequent reactions of hydrocarbons with O_2(g)_ contained in the air, generating CO_2(g)_ and H_2_O_(g)_ as final reactions products. About 97.0% of sample is volatilized at 700 °C for a 15 min dwelling time of the P-PVC sample under air.

The curve shape of W-PVC treatment in the air atmosphere (Figure 7b) is fairly comparable to that of P-PVC but the obtained values of mass loss are quite different. Only 45.9% of the PVC waste sample was lost at 300 °C. Furthermore, the SEM-EDS analysis indicated that there is still chlorine in the resulting residue. This is probably due to the presence of mineral fillers and that a small part of chlorine (evolved mostly as HCl) may have reacted with calcium carbonate which is non-volatile and remained in the treatment residue. The percentage of ML at higher temperatures is also lesser than that of the P-PVC sample due to the relative lower amount of hydrocarbon in this PVC sample. From 700 °C, the sample loss remained almost stable and around 69% ML was obtained at 900 °C. Data of infrared spectroscopy of the W-PVC treatment residues were previously reported [33]. In the IR spectra, between 2950 cm^−1^ and 2840 cm^−1^, C-H elongation signals characteristics of CH_x_ groups that were distinguished; their intensity decreased with increasing temperature and disappeared at T ≥ 600 °C. This seems to be in agreement with the data provided in Figure 7b demonstrating that the mass loss almost stabilized; that is, the hydrocarbon matter had already fully reacted with oxygen at a temperature higher than 600 °C.

The thermal behavior of the clay sample under the same conditions as those used for the PVC samples is described in Figure 7c. After a small mass loss at a low temperature, a significant percentage of ML was observed at a temperature higher than 400 °C, reaching a plateau of about 8.9% at T > 750 °C. Results of the XRD analysis and early findings [34,35] seem to indicate that a full de-hydroxylation of kaolinite is achieved at T < 700 °C. Knowing that the relative mass of water in pure kaolinite [Al_2_Si_2_O_5_(OH)_4_, i.e., Al_2_O_3_·2SiO_2_·2H_2_O] is 13.96%, one may suggest that the kaolinite content in the clay sample is close to 57%.

**Figure 8:**
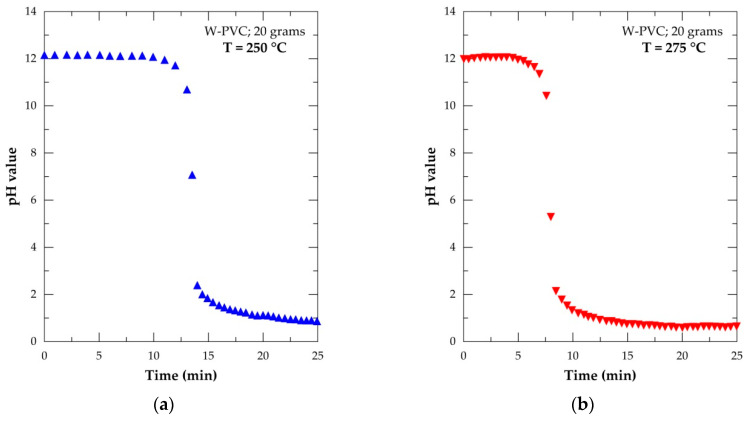
The pH value evolution versus the time of a solution scrubbing the outlet gas treatment of 20 g of W-PVC in the air atmosphere: (**a**) treated at 250 °C and (**b**) treated at 275 °C.

Another investigation considered during the investigation of the thermal treatment of initial samples was to demonstrate the release of HCl when PVC waste is heated at a given temperature. To validate this assumption, two experimental tests were performed at 250 °C and 275 °C using 20 g of the W-PVC sample. The carrier gas was air and the outlet gases were scrubbed in 400 mL of the NaOH solution, having an initial pH equal to twelve. The pH evolution of the solution was recorded versus the time and the results are shown in Figure 8. During the W-PVC treatment at 250 °C (Figure 8a), the value of pH remained almost constant for the first 10 min. This is due to the time required for reaching a thermal equilibrium as well as the time required for gases to travel through the circuit to reach the NaOH solution. After that, a sudden drop in pH value is observed and after 25 min of W-PVC treatment at 250 °C, the solution pH is equal to 0.86. This is an indirect proof of the formation of an aqueous solution of gaseous hydrogen chloride, resulting in strong hydrochloric acid. A similar trend in the evolution of pH versus time is valid for the treatment at 275 °C (Figure 8b), although this so-called “latent period” is shorter and the solution pH after 25 min of treatment was equal to 0.72. This experimental evidence appears to confirm that the rapid kinetics of PVC de-chlorination at temperatures of 275 °C and that the hydrogen chloride is the gaseous reaction product that can be considered as a source for an economical hydrochloric acid production. The results obtained with the individual thermal treatment of the initial reagents will be helpful to follow the thermal behavior of the pellet mixtures composed of hematite, PVC, and clay.

### 3.3. Treatment of (Hematite + PVC + Clay) Mixtures in Various Conditions

**Figure 9:**
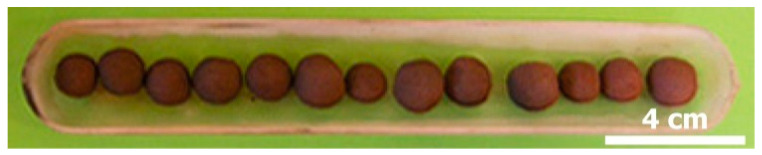
Optical image of pellets (35% hematite + 35% W-PVC + 30% clay) treated at 300 °C for 30 min.

According to results of the previous section, it was suggested to check the capability of PVC to reduce iron oxides by applying the following procedure: (i) heating pellet mixtures of PVC, hematite, and clay for PVC de-chlorination, and (ii) heating obtained products at various temperatures and reaction times. Good numbers of pellets (hematite + PVC + clay) were prepared and then heated at 300 °C under air. Figure 9 is a picture of the pellet mixtures shown in Figure 1 after being heated at 300 °C for 30 min.

The pellets maintained their shapes after this treatment (see Figure 1 and Figure 9 for comparison) and no obvious reaction was observed between the pellet constituents. One may emphasize that hematite is thermodynamically stable in gaseous hydrogen chloride as their interaction (Equation (1)) proceeds with a value of standard free energy changes (Δ_r_G°) of 21.1 kJ/mol at 300 °C [36]. Note that the low reactivity of hematite towards chlorine in a relatively wide temperature range was also observed early [37,38,39,40,41].

1/6 Fe_2_O_3(s)_ + HCl_(g)_ → 1/3 FeCl_3(g)_ +1/2 H_2_O_(g)_ (1)

A series of thermal treatment tests were conducted with de-chlorinated pellets mixtures from 600 °C to 1100 °C with a duration time fixed at 30 min. These tests were performed without gas circulation; in other words, as soon as the boat with the sample is placed in the center of the furnace, the gas inlet (Figure 2) is closed, while the gas-outlet is directed to the suction hood.

**Figure 10:**
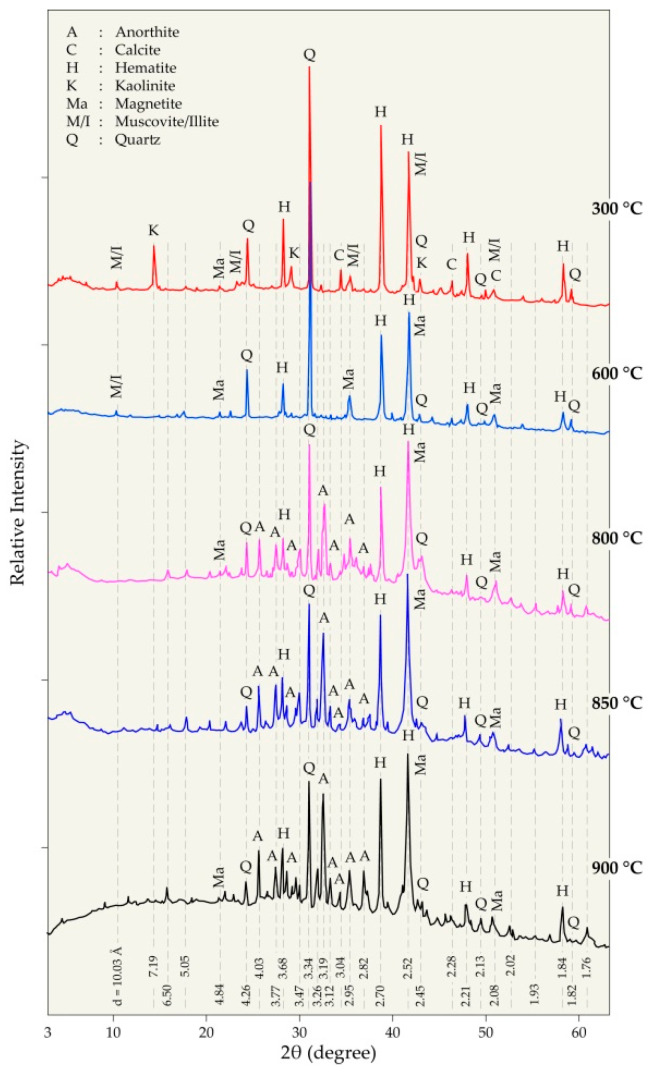
XRD diffractograms of the residues obtained during the treatment of pellets from 300 °C to 900 °C at 30 min.

**Figure 11:**
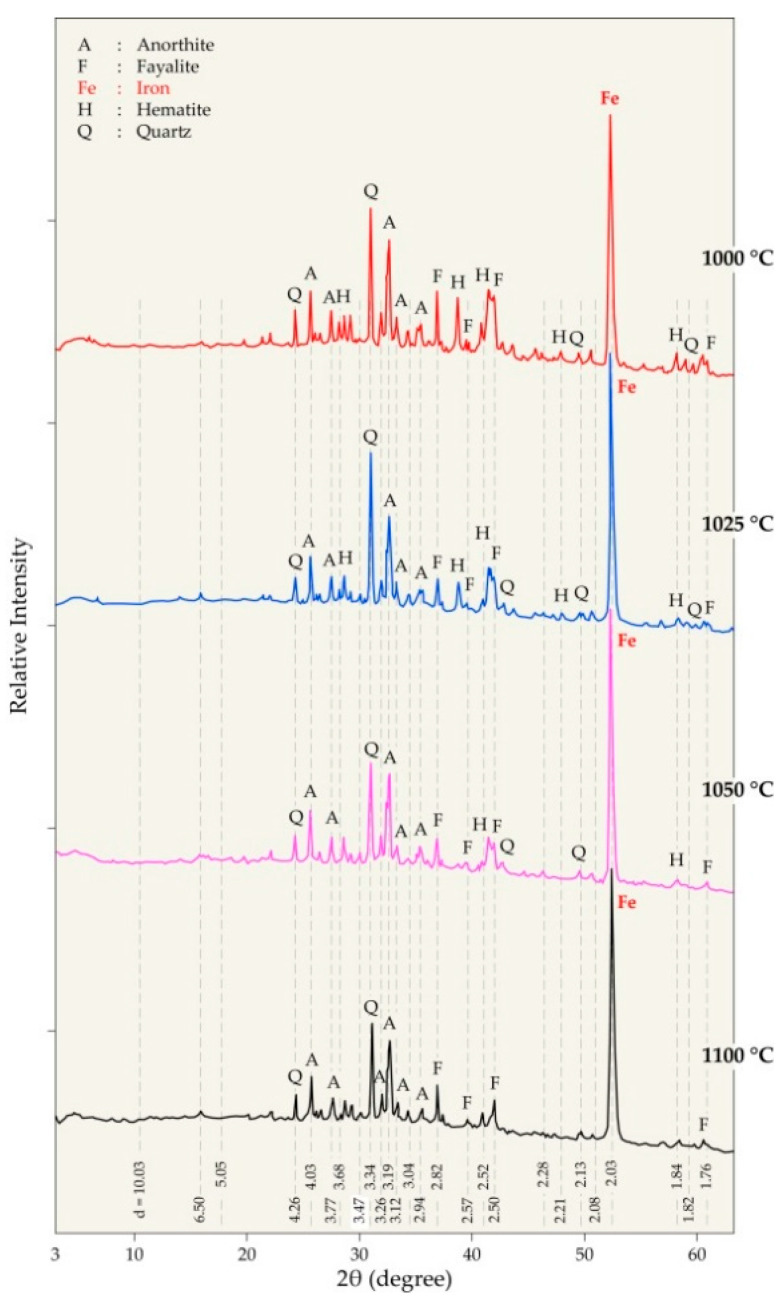
XRD diffractograms of the residues obtained during the treatment of pellets from 1000 °C to 1100 °C at 30 min.

To obtain a basis for the understanding of the thermal behavior of pellets constituents and their interactions, the treatment products were systematically examined by the XRD technique. Figure 10 and Figure 11 summarize the XRD patterns of the residues at 300–900 °C and 1000–1100 °C, respectively.

The XRD pattern of pellet mixtures heated at 300 °C (Figure 10) presents roughly all the phases found in the constituents of the initial mixture including calcite, hematite, kaolinite, micas (muscovite/illite), and quartz. At 600 °C, the characteristic peaks of kaolinite disappeared as they were converted to metakaolin with a disordered structure. Water is evolved during the dehydroxylation of kaolinite according to Equation (2). At this temperature, magnetite (Fe_3_O_4_) appeared in the diffractogram of the treated pellets resulting from the hematite reduction with the hydrocarbons issued from the thermal treatment of the PVC. The well crystallized anorthite (CaAl_2_Si_2_O_8_) phase is identified, beginning from 800 °C. It is synthetized by the reaction of CaCO_3_ contained in the PVC, with SiO_2_ and Al_2_O_3_ originating from the kaolinite sample according to an overall reaction described in Equation (3) and having a value of Δ_r_G° = −118.97 kJ/mol at 800 °C. The synthesis of anorthite from its simple constituents is also mentioned in the literature [42,43].

Al_2_Si_2_O_5_(OH)_4(s)_ → Al_2_Si_2_O_7(s)_ + 2H_2_O_(g)_ (2)

CaCO_3(s)_ + 2SiO_2(s)_ + Al_2_O_3(s)_ → CaAl_2_Si_2_O_8(s)_ + CO_2(g)_ (3)

The increase in temperature at 850 °C and 900 °C for the thermal treatment of pellets at 30 min did not lead to any significant qualitative change of crystallized phases; anorthite, hematite, magnetite, and quartz are still predominant phases of both residues (Figure 10). Micas (muscovite and illite) was no longer detected due to the breakdown of their structure at high temperatures.

The analysis of the XRD diffractograms for the residues obtained at high temperatures (Figure 11) confirmed the presence of metallic iron at all treatment temperatures between 1000 °C and 1100 °C. Anorthite and quartz are also the main phases in the products, while only a weak peak of hematite is detectable in the diffractograms. Wüstite (FeO) is not revealed by XRD, however the fayalite (Fe_2_SiO_4_) induces a predominant phase in the residues.

**Figure 12:**
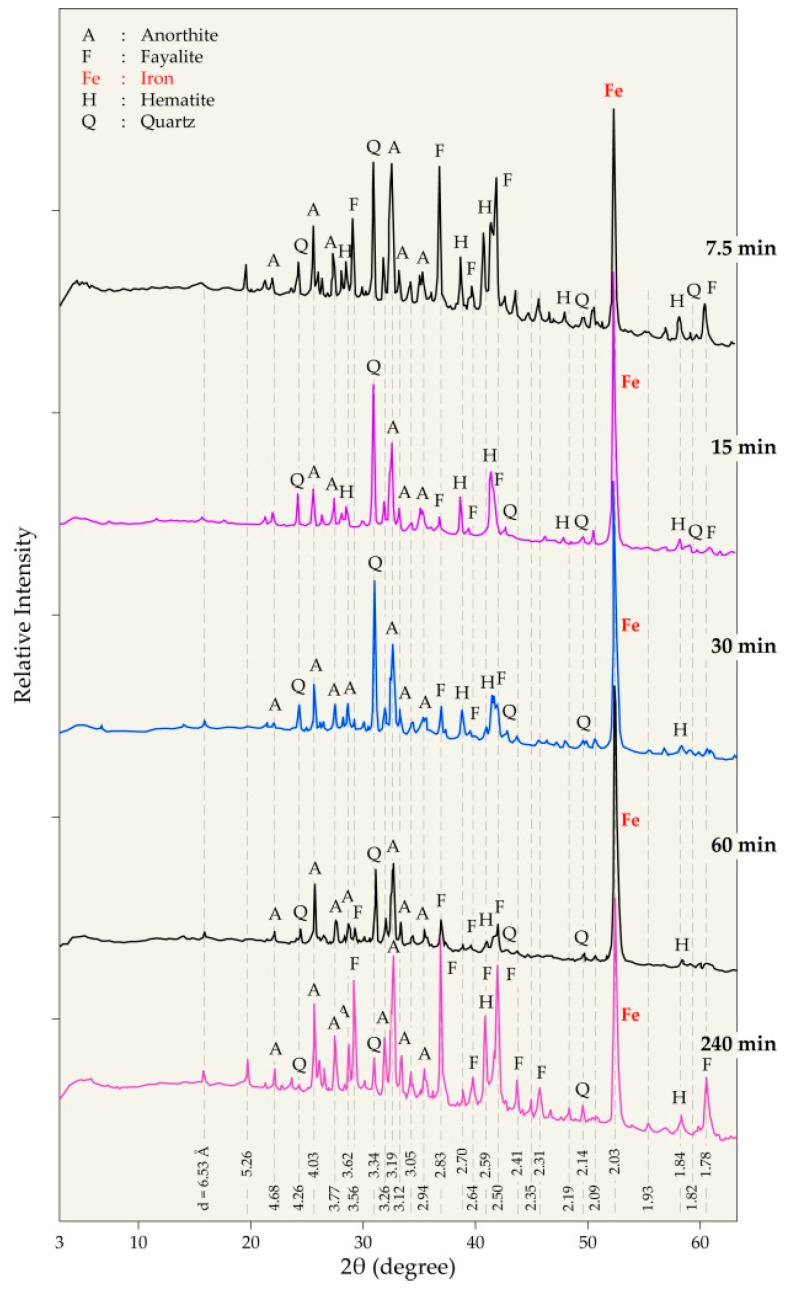
XRD diffractograms of the residues obtained during the treatment of (hematite + W-PVC + clay) the mixture at 1025 °C for different reaction times.

The effect of the reaction time on the phase composition of the pellet mixtures heated at 1025 °C is provided in Figure 12. The formation of metallic iron is clear for a 7.5 min dwelling time at this temperature. Similarly, anorthite and fayalite are synthetized for a short time indicating the high reactivity of their constituents. The increase in processing time did not result in significant differences in the composition of the residues except for the 4 h processing time for which there are more developed peaks for the fayalite phase.

**Figure 13:**
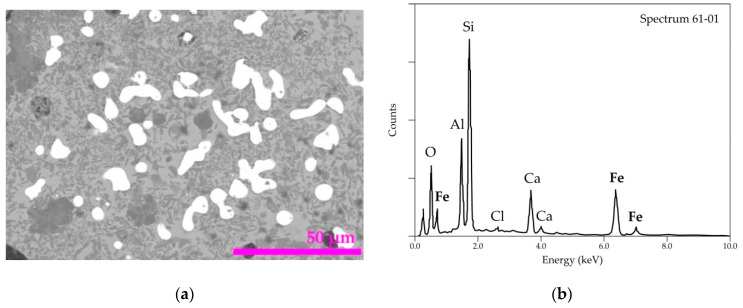
SEM-EDS results of the residue obtained from the treatment of the pellets at 1025 °C for 1 h: (**a**) general view of the obtained residue and (**b**) overall EDS analysis of the residue sample.

It was deemed interesting to investigate the morphological aspects and punctual composition of the residues using the SEM-EDS. Figure 13a shows a SEM image of the cross-section of a pellet treated at 1025 °C for 60 min. The difference in the phase contrast is obvious in the SEM image. Furthermore, the rounded shape of the almost-white particles suggests that a liquid phase was formed during the treatment. The general EDS spectrum provided in Figure 13b displays the main elements of the analyzed areas. Note that the identification of chlorine that is probably associated with calcium (as CaCl_2_) synthetized during the de-chlorination process and remained in the residue.

**Figure 14:**
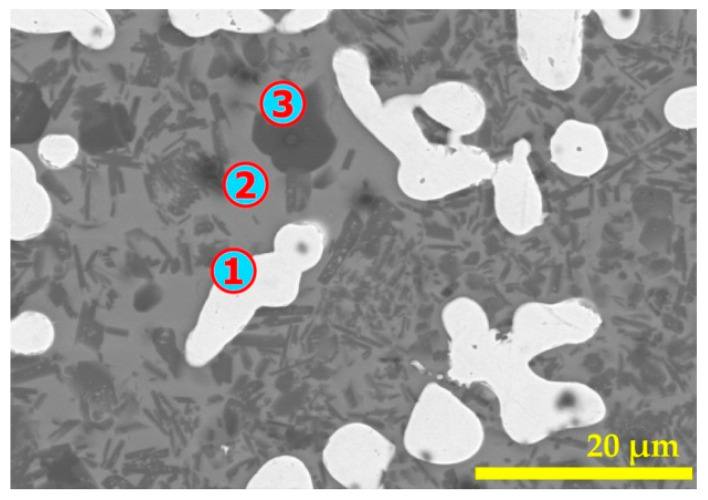
SEM aspects (BSE image) of the pellets treated at 1025 °C for 1 h. The numbers 1 to 3 indicate the locations of microanalysis.

More detailed results (SEM-EDS analysis) for this residue are grouped in Figure 14 and Table 2. The white, grayish, and dark areas of the SEM images are analyzed by EDS for determining their elemental composition. The EDS analysis of spot n° 1 revealed that iron is the only constituent of this area. As carbon is considered, one may suggest that this white area may also contain carbon which can justify the fusion of these particles to some extents as the melting temperature of iron is 1538 °C [36], whereas in the presence of carbon, the melting point of this material (pig iron) may drop by about 300 °C. Spot n° 2 represents a mixed zone containing (Si, Fe, Al, and Ca) oxides. Silicon oxide (quartz) consisted in mainly the zone represented by spot n° 3.

**Figure 15:**
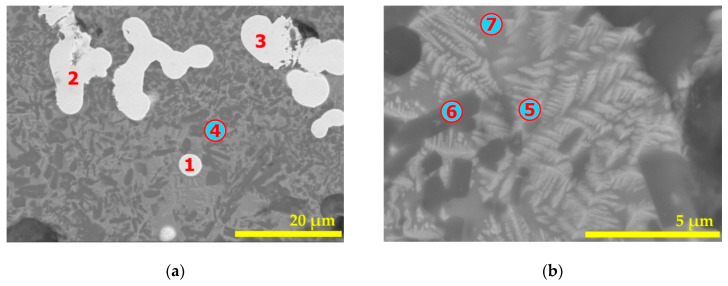
SEM micrographs (BSE image) of the (hematite + W–PVC + clay) sample treated at 1025 °C for 2 h: (**a**) general view of the obtained residue and (**b**) greater magnification in an area having a complex composition. The numbers 1 to 7 indicate the locations of microanalysis.

Figure 15 and Table 3 group the results for a residue obtained after 2 h of treatment at 1025 °C. As in the previous case, the white areas (locations n° 1, 2, and 3) are composed of iron with probably some amount of carbon. The area with microanalysis of spot n° 4 appeared to be anorthite (CaAl_2_Si_2_O_8_) with some substitution of aluminum by ferric iron. Spot n° 6 (Figure 15b) appeared to have a comparable composition to that of spot n° 4. Microanalyses of locations n° 5 and n° 7 indicate an iron silicate when some ferrous iron is substituted with calcium. Their composition contains excess silicon and is attributed to a fayalite phase.

**Figure 16:**
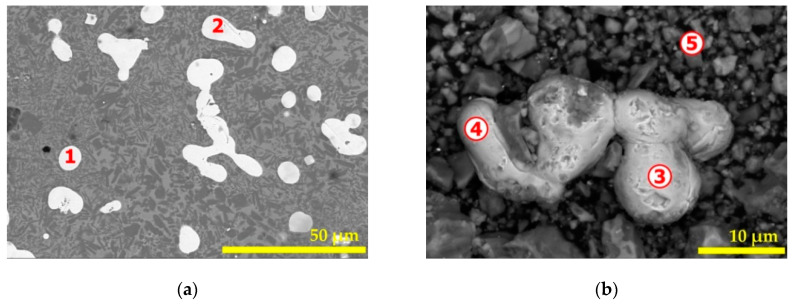
SEM aspects of the (hematite + W-PVC + clay) sample treated at 1025 °C for 4 h: (**a**) BSE image of the polished surface sample and (**b**) BSE image of the powdered sample. Numbers 1 to 5 indicate the locations of microanalysis.

To obtain more consistent information about the morphology of the reduced hematite by de-chlorinated PVC at 4 h, a powdered sample was examined also by SEM-EDS. The SEM image set (polished and powdered specimen) are displayed in Figure 16. In both cases, BSE images were favored for a rapid and clearer phase discrimination. Results of EDS-microanalysis are reported in Table 4 and comments about the particle composition are comparable with those provided in previous paragraphs. The image in Figure 16b indicates the shape of almost pure and dense iron particles in a size magnitude of tens of micrometers. As the initial hematite sample was constituted of micrometric particles, a possible sintering of the material occurred through the aggregation of iron particles. The well identified iron segregates presenting clear boundaries from the rest of the sample will be useful for their subsequent separation by physical methods.

**Figure 17:**
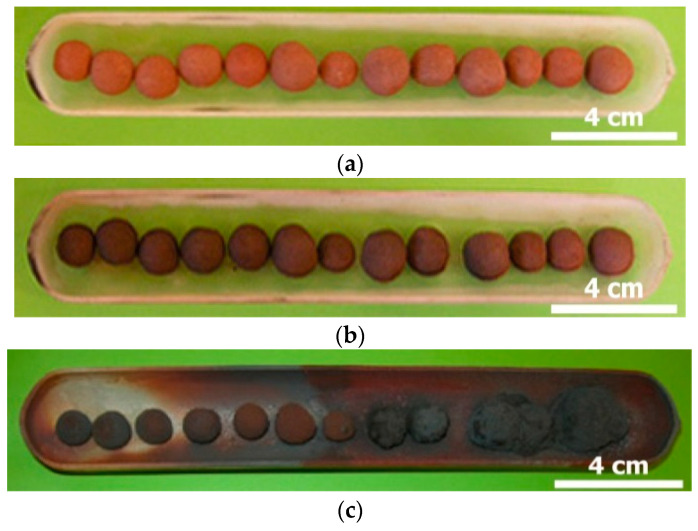
Visual images of (hematite–PVC–kaolinite) mixtures: (**a**) initial mixture; (**b**) treated at 300 °C; and (**c**) treated at 1050 °C.

Another series of experimental tests were conducted at 1050 °C using pellets of the same composition (i.e., 35% hematite + 35% W-PVC + 30% clay). In these trials, air with a flow rate of 2.5 L/h was used. Figure 17 displays the boat with the pellets at the initial state, (a) treated at 300 °C and (b) 1025 °C (c) in an air atmosphere for 30 min. It is obvious that most of the pellets shrunk after their treatment at 1050 °C. In addition, those in front of the air inlet are twinned and merged probably due to the high exothermic nature of the reactions of oxygen with hydrocarbon and carbonaceous materials (char). The shrinkage of the pellets was observed from 1000 °C in almost all experimental tests and due probably to particle sintering and/or to the fusion of some components of the pellets. It is believed that the shrinking of the pellets allowed for good contact of oxides with the reducing agents, thus improving the reduction process.

**Figure 18:**
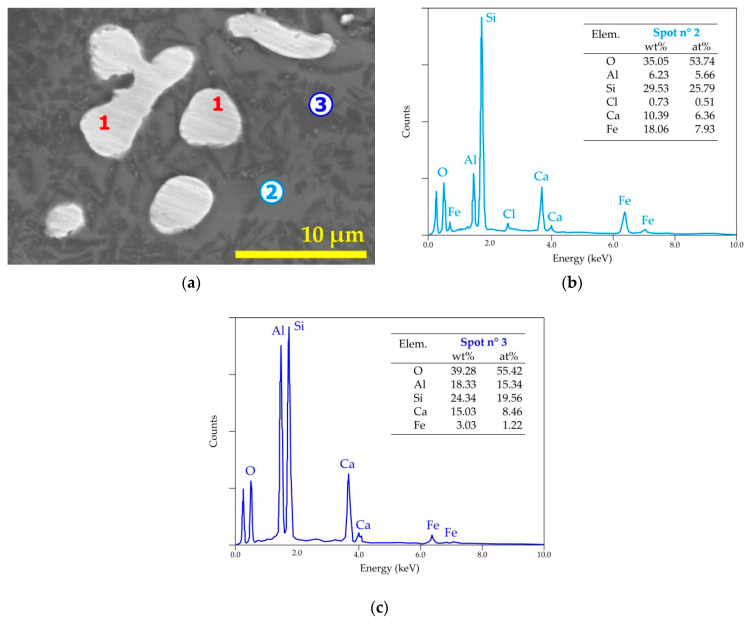
SEM-EDS results of the (hematite + W-PVC + clay) sample treated at 1050 °C for 30 min in the air atmosphere: (**a**) general view (BSE micrograph) of the obtained sample; (**b**) EDS analysis of spot n° 2; and (**c**) EDS analysis of spot n° 3.

Several SEM-EDS results of the residue derived from the thermal treatment in the presence of air and reported in Figure 18 indicated similar trends to those exposed for the treatment without gas circulation. The white areas indicated by n° 1 (Figure 18a) are almost dense iron particles. The greyer area n° 2 is a mixed zone with a complex composition of aluminosilicate of calcium and iron. The darker zone with the punctual analysis of spot n° 3 seems to reflect a mixed phase of the anorthite and fayalite species.

Thus, through these experimental and analytic results, one may hypothesize the possible chemical reactions of the complex system for the hematite reduction of the studied pellet mixture containing considerable amounts of silicon, aluminum, and calcium-bearing compounds.

Although the carbon of the carbonaceous char should be a predominant reducing agent for the direct reduction of iron oxides, their general reduction scheme (Fe_2_O_3_ → Fe_3_O_4_ → FeO → Fe) should be achieved through intermediate species synthetized in the reaction zone during the thermal treatment. The values of standard entropy (ΔrH°) and free energy (ΔrG°) changes at 1000 °C computed using the HSC thermochemical database [36] are provided below (Table 5) for a set of reactions (Equations (4)–(20)) likely to take place during the thermal treatment and considering simple reducing agents such as C_(s)_, CO_(g)_, and H_2(g)_. Several scientific findings devoted to the thermodynamic and kinetics aspects as well as the modeling of iron oxide reductions with various reducing agents were summarized recently [44,45,46,47,48]; most of their developed approaches were based on selected reactions being analogous to those described by Equations (4)–(20).

Reactions of the direct reduction of iron oxides by solid carbon are of endothermic nature (Equations (4)–(6)), hence they should be favored at high temperatures. A similar deduction is valid for the gasification of carbon according to Equation (12), known as the Boudouard reaction, and through the straightforward water–gas reaction described by Equation (13). The resulting reducing gases of CO and H_2_ will then intervene in the reduction of iron oxides according to Equations (7)–(9) and (15)–(17), respectively. The source of water can be attributed to the dehydroxylation of kaolinite and to some extents to the structural water of micas. As shown in Figure 17, the assumed partial fusion of the pellets (pellet contraction) will probably enhance the surface contact of reagents and create a barrier preventing the rapid escape of gases, leading to a longer contact time between the iron oxides and reactive species. This is one hypothesis explaining the increase of temperature in the reaction zone (although unknown), leading to the fusion of metallized iron and/or its alloys.

Another important point of the study is the possibility of the interaction between basic (CaO) and acid (SiO_2_ and Al_2_O_3_) oxides (Equations (3) and (18)) at relatively low temperatures to forms stable compounds such as anorthite. However, if the silica content is high, more basic oxides should be added in order to prevent the formation of fayalite (Equation (19)) and/or to release wüstite according to Equation (20) before its reduction to metallic iron.

## 4. Conclusions

PVC waste can be considered as a carbonaceous source useful as an energetical and reducing agent for pyrometallurgical processes (ferrous industry), thus attractive in the context of a circular economy. As the presence of chlorine leads to difficulties in its direct uses in industrial units, a two-step process should be an alternative solution for the valorization of the PVC waste in the direct reduction materials bearing iron oxide. The following points can be drawn from this study:

The de-chlorination of PVC can be achieved at temperatures near 300 °C and the kinetic of the chlorine release as HCl is fast at temperatures close to 275 °C. Small amounts of chlorine can be retained during the treatment of PVC waste probably due to its reactions with calcium carbonate found in the end-of-life of PVC materials.

The constituents of the prepared pellets (hematite, PVC waste, and clay) underwent a series of transformations during thermal treatment between 600 °C and 1100 °C. The reduction of hematite into magnetite is observed from 600 °C and both phases coexist up to at least 900 °C. Kaolinite de-hydroxylation generates a vapor stream; this last phase can react with char carbon, producing hydrogen and carbon monoxide as the intermediate species for reducing iron oxides.

The gangue oxides (CaO, Al_2_O_3_, and SiO_2_) interacted by synthetizing anorthite (CaAl_2_Si_2_O_8_), a stable phase at all treatment temperatures up to 1100 °C. The thermal treatment at 1000 °C and above led to the metallization of iron oxides. Wüstite is not observed due to the high reactivity towards quartz, leading to the formation of fayalite. This should be avoided by increasing the CaO and Al_2_O_3_ amount in the used material permitting a stable anorthite synthesis.

Dense, enlarged, and segregated grains of metallic iron and/or pig iron were obtained during the pellet thermal treatment at 1025–1050 °C that allows their subsequent separation to be easy and thus appropriate for steel production.

This study demonstrates some important aspects of the behavior of iron oxides in a complex system during the valorization of PVC waste based on the microanalysis results. More kinetics aspects must be investigated for the clarification of the intrinsic reaction mechanisms governing the thermal process. Furthermore, the processing of mixed plastics and mineral compounds issued from automobile shredder residues are in progress and will be the topic of a companion paper.

## Figures and Tables

**Table 1:** Elemental composition of W-PVC analyzed by SEM-EDS.

Elements	Spot n° 1		Spot n° 2		Spot n° 3	
-	^1^ wt%	^1^ at%	wt%	at%	wt%	at%
O	7.35	22.26	55.74	75.13	7.64	17.88
Al	2.18	3.92	0.60	0.48	5.27	7.32
Si	2.38	4.11	0.73	0.56	5.61	7.47
Cl	40.52	55.40	10.45	6.36	56.83	60.01
Ca	2.03	2.45	32.48	17.48	1.73	1.62
Sb	7.32	2.92	-	-	12.31	3.78
Pb	38.22	8.94	-	-	10.61	1.92

^1^ wt% and at% represent mass and atomic percentage, respectively.

**Table 2:** Elemental composition (EDS data) of the pellets treated at 1025 °C for 1 h.

Elements	Spot n° 1		Spot n° 2		Spot n° 3	
	^1^ wt%	^1^ at%	wt%	at%	wt%	at%
O	-	-	36.97	57.68	48.30	62.11
Al	-	-	4.51	4.17	0.78	0.59
Si	-	-	23.91	21.25	50.93	37.30
Cl	-	-	0.92	0.65	-	-
Ca	-	-	6.72	4.19	-	-
Fe	100.00	100.00	26.97	12.06	-	-

^1^ wt% and at% represent mass and atomic percentage, respectively.

**Table 3:** Elemental composition (EDS data) of the (hematite + W-PVC + clay) sample treated at 1025 °C for 2 h.

Elements	Spot n° 1,2,3		Spot n° 4		Spot n° 5		Spot n° 6		Spot n° 7	
	^1^ wt%	^1^ at%	wt%	at%	wt%	at%	wt%	at%	wt%	at%
O	-	-	40.05	57.03	34.03	55.91	42.47	59.19	35.51	56.00
Al	-	-	16.35	13.81	3.31	3.22	12.58	10.40	4.42	4.14
Si	-	-	22.71	18.43	22.61	21.16	26.67	21.17	24.36	21.88
Cl	-	-	-	-	-	-	-	-	0.75	0.54
Ca	-	-	13.76	7.82	4.63	3.04	12.33	6.86	9.28	5.84
Fe	100.00	100.00	7.13	2.91	35.42	16.67	5.95	2.38	25.68	11.60

^1^ wt% and at% represent mass and atomic percentage, respectively.

**Table 4:** Elemental composition (EDS data) of the (hematite + W-PVC + clay) mixture treated at 1025 °C for 4 h.

Elements	Spot n° 1,2		Spot n° 3		Spot n° 4		Spot n° 5	
	^1^ wt%	^1^ at%	wt%	at%	wt%	at%	wt%	at%
O	-	-	3.15	9.91	3.17	10.01	30.89	49.36
Al	-	-	0.69	1.29	1.08	2.02	11.09	10.51
Si	-	-	2.43	4.35	1.36	2.46	25.81	23.49
Ca	-	-	-	-	-	-	10.52	6.71
Fe	100.00	100.00	93.73	84.45	94.39	85.51	21.70	9.93

^1^ wt% and at% represent mass and atomic percentage, respectively.

**Table 5:** Selected reactions and thermodynamic data of iron oxide reductions with various reducing agents.

Chemical Reactions	Δ_r_H°_1000 °C_ (kJ)	Δ_r_G°_1000 °C_ (kJ)	Equation Number
3 Fe_2_O_3(s)_ + C_(s)_ → 2 Fe_3_O_4(s)_ + CO_(g)_	125.70	−171.97	(4)
Fe_3_O_4(s)_ + C_(s)_ → 3 FeO_(s)_ + CO_(g)_	181.76	−56.08	(5)
FeO_(s)_ + C_(s)_ → Fe_(s)_ + CO_(g)_	152.09	−43.28	(6)
3 Fe_2_O_3(s)_ + CO_(g)_ → 2 Fe_3_O_4(s)_ + CO_2(g)_	−42.05	−119.70	(7)
Fe_3_O_4(s)_ + CO_(g)_ → 3 FeO_(s)_ + CO_2(g)_	14.016	−3.81	(8)
FeO_(s)_ + CO_(g)_ → Fe_(s)_ + CO_2(g)_	−15.65	8.99	(9)
2 C_(s)_ + O_2(g)_ → 2 CO_(g)_	−227.42	−448.39	(10)
C_(s)_ + O_2(g)_ → CO_2(g)_	−395.17	−396.12	(11)
C_(s)_ + CO_2(g)_ → 2 CO_(g)_	167.74	−52.27	(12)
C_(s)_ + H_2_O_(g)_ → CO_(g)_ + H_2(g)_	135.55	−46.69	(13)
CO_(g)_ + H_2_O_(g)_ → CO_2(g)_ + H_2(g)_	−32.20	5.58	(14)
3 Fe_2_O_3(s)_ + H_2(g)_ → 2 Fe_3_O_4(s)_ + H_2_O_(g)_	−9.85	−125.27	(15)
Fe_3_O_4(s)_ + H_2(g)_ → 3 FeO_(s)_ + H_2_O_(g)_	46.21	−9.40	(16)
FeO_(s)_ + H_2(g)_ → Fe_(s)_ + H_2_O_(g)_	16.54	3.42	(17)
1/2 CaO_(s)_ +SiO_2(s)_ + 1/2 Al_2_O_3(s)_ → 1/2 CaAl_2_Si_2_O_8(s)_	−58.26	−66.95	(18)
2 FeO_(s)_ + SiO_2(s)_ → Fe_2_SiO_4(s)_	−40.04	−13.62	(19)
2 Fe_2_SiO_4(s)_ + CaO_(s)_ + Al_2_O_3(s)_ → CaAl_2_Si_2_O_8(s)_ + 4 FeO_(s)_	−37.67	−92.80	(20)

## Data Availability

The data presented in this study are available upon request to the corresponding author.
